# Refining the LE8-multimorbidity-af nexus: methodological considerations for enhanced clinical translation

**DOI:** 10.1097/JS9.0000000000003113

**Published:** 2025-07-23

**Authors:** Yidan Dong, Mingzhu Wang, Deyu Fu

**Affiliations:** Yueyang Hospital of Integrated Traditional Chinese and Western Medicine, Shanghai University of Traditional Chinese Medicine, Shanghai, China

*Dear Editor*,

We read with great interest the recent article by Junguo Zhang *et al* titled “The Role of Life’s Essential 8 and Multimorbidity in the Risk of New Onset Atrial Fibrillation: Observations from a Large Prospective Cohort Study”^[1]^. The authors conducted an association analysis examining the relationship between healthy lifestyle behaviors, as defined by Life’s Essential 8 (LE8), various categories of 36 long-term diseases, and the risk of atrial fibrillation. They made preliminary observations regarding the association between LE8 scores, individual or multiple comorbid conditions, and the incidence of atrial fibrillation. Through interaction and mediation analyses, the authors provided a thorough exploration that offers valuable guidance for clinical treatment and prevention. Although the article acknowledges certain limitations of the research, we believe that the following aspects warrant further discussion, as they may influence the interpretation of the current findings and inform future research directions. We confirm this commentary adheres to the TITAN 2025 methodological standards^[2]^, with no artificial intelligence tools used in its development.

Firstly, the exclusion criteria for the study only considered individuals with a history of atrial fibrillation. However, additional medical histories—including heart disease, valvular disorders, and previous cardiac surgeries—should also be evaluated, as these factors may significantly impact the incidence and long-term outcomes of atrial fibrillation^[3]^, this limitation could compromise the reliability of the results. Furthermore, the diagnostic criteria for atrial fibrillation require clarification. Have the physicians’ diagnoses recorded in primary care databases, hospitalization records, and death registries been confirmed using a standardized and clear diagnostic protocol? Additionally, a broader range of atrial fibrillation types should be explicitly defined and considered in the analysis.

Secondly, there is insufficient consideration of confounding factors. The eight indicators in Life’s Essential 8 (LE8) are primarily analyzed as continuous variables; however, stratifying them by categorical variables, such as obesity classification based on body mass index—normal, overweight, and lean—could yield a clearer understanding of disease risk^[4]^. Aging is also a major risk factor for atrial fibrillation. Studies indicate a significant increase in the risk of atrial fibrillation among the elderly, making stratified analysis by age necessary^[5]^, especially given the research’s emphasis on the protective effects of youth and female gender on cardiovascular health. Changes in menopausal hormone levels after aging are also critical factors influencing the occurrence of atrial fibrillation in women^[6]^. Additionally, differences in race and ethnicity may contribute to varying disease trends^[7]^. Although the discussion section acknowledges the need for further consideration of confounding factors, it is important to confirm that various social and psychological stressors, venous thromboembolism, chronic kidney disease, and other factors can significantly elevate the risk of atrial fibrillation. The mechanisms by which these confounding variables may trigger atrial fibrillation include complex pathways involving neuroendocrine activation, oxidative stress, endothelial dysfunction, inflammation, and atrial fibrosis^[8]^.

Finally, the comorbidities of diabetes and hypertension mentioned in the study appear to overlap with the key indicators in the LE8 score. This overlap may lead to repeated effects in the interaction and combination of associations, potentially compromising the accuracy of the conclusions drawn. Additionally, the presence of atrial fibrillation is confirmed by monitoring subjects without a history of AF until new cases of AF arise, or until the subjects experience death, loss to follow-up, or the conclusion of the follow-up period. In observational cohort studies, the earlier occurrence of other diseases, aside from atrial fibrillation, may hinder the investigation of the true association of lifestyle scores, cardiovascular health, and the incidence of atrial fibrillation. Employing a Fine-Gray competing risk model could more accurately assess disease risk and draw universally applicable conclusions^[9]^.

In summary, the interpretation of the relevant results should be approached with caution. More statistically robust analysis methods should be utilized to evaluate the associations of lifestyle, comorbidity status, and the incidence of atrial fibrillation, particularly regarding their interactions and joint effects, which would help avoiding misleading conclusions that are challenging to generalize for clinical applications.

## Data Availability

Data availability is not applicable to this article as no new data were created or analyzed in this study.
